# Attitudes, Norms, and Control: What Is Shaping Fijian Children's Physical Activity and Screen Time Behaviours?

**DOI:** 10.1111/cch.70256

**Published:** 2026-03-18

**Authors:** Sarah T. Ryan, Anthony D. Okely, Rebecca M. Stanley, Gade Waqa, Melanie Randle

**Affiliations:** ^1^ Early Start University of Wollongong Wollongong Australia; ^2^ School of Health and Society, Faculty of the Arts, Social Sciences and Humanities University of Wollongong Wollongong Australia; ^3^ C‐POND, College of Medicine, Nursing and Health Sciences Fiji National University Suva Fiji; ^4^ School of Business, Faculty of Business and Law University of Wollongong Wollongong Australia

## Abstract

**Background:**

Only half the children in Fiji meet both physical activity (PA) and screen time (ST) recommendations. Understanding factors associated with meeting these recommendations is important to inform the development of policies and programmes to encourage healthy behaviours. This study aimed to examine the association between Fijian caregivers' and children's attitudes, subjective norms and perceived behavioural control and their or their child's adherence to physical activity and screen time guidelines.

**Methods:**

We investigated Fijian caregivers and children aged 5–17 years attitudes, subjective norms and perceived behavioural control towards meeting PA and ST guidelines through an online survey. The survey explored these factors and their associations with meeting the Asia‐Pacific Integrated 24‐h Activity Guidelines for Children and Adolescents.

**Results:**

A total of 1015 caregivers and 699 of their children completed the survey. Caregivers (OR = 1.4, CI 1.003–1.862) and children 5–8 years (OR = 16.5, CI 1.227–222.665) who believed being active would make them happier were more likely to meet PA recommendations compared to those not meeting PA recommendations. Caregivers who set ST restrictions (OR = 1.5, CI 1.252–1.816) and believed ST rules helped their child meet recommendations (OR = 1.3, CI 1.052–1.505) were more likely to have children who met ST recommendations versus not meet ST recommendations. Children aged 5–8 and older children/adolescents aged 9–17 who had easy access to screens were less likely to meet ST recommendations (OR = 0.5, CI 0.224–0.984) and (OR = 0.5, CI 0.324–0.802), respectively, compared to those who did meet ST recommendations. Children aged 5–8 years who believed it was important to follow the guidance of religious leaders were more likely to meet ST recommendations (OR = 5.4, CI 2.423–12.002) compared to those who did not meet the ST recommendation.

**Conclusion:**

Communicating through trusted community figures (such as teachers and religious leaders for younger children and ministries for caregivers) is recommended for future initiatives to increase adherence to guidelines among children and adolescents in Fiji. These initiatives should also emphasise the link between PA and both happiness and academic performance, while exploring ways to support caregivers in enforcing ST restrictions.

## Key Points

- Identifies key factors influencing physical activity and ST behaviours among Fijian children/adolescents and caregivers.
- Highlights the potential of teachers and religious leaders as trusted messengers for younger children, whereas caregivers place greater trust in ministries and schools.
- Provides evidence‐based recommendations for tailoring social marketing campaigns to promote healthy behaviours in Fijian children.
- Emphasises the importance of addressing age‐specific motivations and barriers in health promotion strategies.
- Offers insights into the unique cultural context of health behaviours in Fiji, informing future public health interventions.

## 1 Background

Fiji has a high prevalence of childhood overweight and obesity (World Obesity Federation 2019, 2023), with rates expected to rise annually by 4.3% between 2020 and 2035. Although nearly 90% of children in Fiji meet physical activity recommendations, only two thirds meet the sedentary recreational screen time (ST) recommendations, and only half meet both the physical activity (PA) and ST recommendations (Ryan et al. 2025). The Pacific Ending Childhood Obesity (ECHO) Network is comprised of 22 Pacific Island Countries and Nations and key organisations such as the World Health Organisation, the Centre for the Prevention of Obesity and Non‐Communicable Diseases (C‐POND) and the Pacific Community (SPC). The Pacific ECHO Network identified promoting physical activity (PA) and reducing recreational sedentary behaviour as a key strategy to reduce the prevalence of childhood obesity in the Pacific and recommended a social marketing campaign (Ravuvu et al. 2021; Pacific Ending Childhood Obesity (ECHO) Network 2019; Pacific Community (SPC) 2019).

### 1.1 Social Marketing in Fiji

Parents or primary caregivers, families and social structures play an important role in shaping children's attitudes, beliefs and behaviours (Lynn Ho et al. 2022). Understanding the perceptions of these stakeholders is essential for developing effective social marketing interventions that target children's behaviour. However, existing social marketing health research in the Pacific Region is limited (Ryan et al. 2022), with few behaviour change interventions applying true social marketing approaches. Many interventions lack the use of a theoretical framework (Stead et al. 2007; Aceves‐Martins et al. 2016). The Global Action Plan on Physical Activity (GAPPA) (World Health Organization 2018) encourages the use of social marketing and best practice communication campaigns to create active societies by focusing on social norms and attitudes. GAPPA promotes these methods to educate people on the numerous benefits of physical activity. However, there are no known data in Fiji that can inform the development of a social marketing campaign, which specifically aims to increase physical activity and reduce sedentary behaviour among children and adolescents.

### 1.2 Theoretical Framework

The Theory of Planned Behaviour (TPB) comprises three belief‐based constructs: attitude, subjective norm and perceived behavioural control, each with two distinct components (Ajzen 1991). Collectively, these three constructs determine the extent to which an individual intends to perform a particular behaviour, with the assumption that intention is the immediate precursor to actual behaviour. The TPB was an appropriate theoretical framework for the present study because it has been successfully applied in child and family behaviour interventions related to PA (Hamilton and White 2012; Rhodes et al. 2021).

We aimed to examine the association between Fijian caregivers' and children's attitudes, subjective norms and perceived behavioural control and their or their child's adherence to physical activity and screen time guidelines. This was guided by the TPB. The findings were used to inform recommendations for a culturally appropriate social marketing campaign to improve the rates of meeting these guidelines among children and adolescents in Fiji.

## 2 Methods

Our research adhered to Improving the Quality of Web Surveys: the Checklist for Reporting Results of Internet E‐Surveys (CHERRIES) for online survey reporting (Eysenbach 2012) (see Table S1). The term ‘children’ is used throughout the manuscript; where relevant, we refer to ‘younger children’ (5–8 years) and ‘older children/adolescents’ (9–17 years) to distinguish age groups.

### 2.1 Ethical Considerations

The study received ethics approval from three institutions: the University of Wollongong (#2019/199), Fiji National University (#175.20) and Fiji's Ministry of Education (#RA 5/22). Informed consent was obtained from both the caregivers and their child, with children providing tacit assent (see Table S1). Data security complied with the Global Data Protection Regulations, utilising REDCap tools hosted at the University of Wollongong (Harris et al. 2009; Harris et al. 2019).

### 2.2 Survey Construction and Refinement

Using the framework of the TPB (Ajzen 2019), our survey explored the factors associated with meeting the Asia‐Pacific Integrated 24‐h Activity Guidelines for Children and Adolescents (World Health Organization 2020; Loo et al. 2022). The Asia‐Pacific guidelines recommend a daily average of 60 min or more of moderate‐ to vigorous‐intensity physical activity (MVPA) and no more than an average of 2 h of sedentary recreational ST per day (Loo et al. 2022). The survey questions were developed with experts in PA and social marketing to ensure alignment of the theoretical constructs and evidence‐based methods for collecting PA data. Local contacts and coauthors (GW and MK) reviewed the survey for cultural appropriateness and suitability to the local context (see Table S1). Item constructs were developed from existing surveys about PA (Nelson et al. 2010), research exploring barriers and facilitators (Minges et al. 2015) and, where possible, specific to the Pacific context (Schluter et al. 2011; Heard et al. 2017).

Caregivers completed the first half of the survey and then were asked if they had a child aged between 5–17 years who could complete the second section. Younger children 5–8 years were provided a shorter version of the survey to ensure comprehension of younger children and to reduce participant burden. A short video explaining the guideline recommendations was provided to the caregiver and the child to assist with comprehension before answering the questions (provided in Table S2). The survey took approximately 10 min for a caregiver and 20 min for a child to complete (separately). The survey was structured to minimise knowledge question bias or contamination.

### 2.3 Recruitment and Data Collection

A comprehensive strategy was implemented across Fiji to recruit a national convenience sample of caregivers and their children aged 5–17 years. The survey period was from July 2023 to April 2024. Initial email contact was made with every school in Fiji (*n* = 912) inviting them to participate in the study and to distribute the survey through their usual communication channels. In addition, we conducted face‐to‐face visits in 61 schools to discuss the survey with the principals. These schools were selected based on a combination of factors, including school size, geographic location and pre‐existing relationships with the research team. The open survey access was distributed to all invited participants via email links and QR codes, supplemented by multilingual promotional videos for social media circulation. Completion of the survey was voluntary. Incentives to encourage participation included school‐level reports for principals and prize draw entries for caregivers, where both caregiver and child survey components were completed. To maximise response rates, a multichannel follow‐up strategy was implemented. This included personalised email and SMS reminders to caregivers, alongside regular communications with schools to foster ongoing support. Approximately 6000 follow‐up emails were sent, averaging 2.8 per respondent. Each respondent also received an estimated five SMS reminders, whereas schools received around 15 emails and 10 SMS/Viber messages encouraging survey support (see Table S1).

### 2.4 Measures

#### 2.4.1 TPB Constructs

##### 2.4.1.1 Attitude

Behavioural beliefs were measured by asking caregivers to rate how important it is that their child is happy, healthy, has friends, and so on. Answers were recorded on a 5‐point Likert scale from −2 (*not at all important*) to +2 (*extremely important*). Evaluation of consequences was measured by asking caregivers whether adhering to PA and ST guidelines would help their child be happier, healthier, make friends, and so on. They answered on a 5‐point Likert scale from −2 (*strongly disagree*) to +2 (*strongly agree*). Children responded to similar statements using a simpler 2‐point scale (*not important/important* and *agree/disagree*). Statements for each participant group can be found in Tables 1, 3 and 4.

##### 2.4.1.2 Subjective Norms

Normative beliefs were measured by asking caregivers to rate whether others (partners, family, friends and religious leaders) think it is important for their child to be physically active or restrict their ST. Answers were recorded on a 5‐point Likert scale from −2 (*strongly disagree*) to +2 (*strongly agree*). Motivation to comply was measured by asking caregivers to rate how much they care about what these people think they should do. They answered on a scale from −2 (*do not care at all*) to +2 (*care very much*). Each question within the subjective norm construct for caregivers had a ‘not applicable to me’ option. Children provided similar ratings tailored to their social context using a simpler 2‐point scale (*disagree/agree and no*, *I do not care/yes* and *I do care*). Statements for each participant group can be found in Tables 1, 3 and 4.

##### 2.4.1.3 Perceived Behavioural Control

Control beliefs were measured by asking caregivers to rate if they had enough money, time, access to transport, among others, to do what they want to do. The answers were recorded on a 5‐point Likert scale from −2 (*no*, *never*) to +2 (*yes*, *always*). Control factor salience was measured by asking caregivers to rate factors (e.g., availability of money, transport and ST restrictions) made it easier or more difficult for their child to meet PA and ST recommendations. The answers were recorded on a 5‐point Likert scale from −2 (*extremely difficult*) to +2 (*extremely easy*). Children provided similar ratings tailored to their social context using a simpler 2‐point scale (*no*, *not important/yes*, *impotent* and *no/yes*). Statements for each group can be found in Tables 1, 3 and 4.

### 2.5 Trust in Institutions/Groups to Provide Health Information

The extent to which caregivers trusted particular agencies and channels to provide health information regarding their children's PA and ST (Ministry of Health, Ministry of Education, Religious Leaders, Social Media) was measured using a 3‐point Likert scale (−1: *disagree*; 0: *neither agree nor disagree*; and +1: *agree*). Statements for each group can be found in Table 2.

### 2.6 Asia‐Pacific Integrated 24‐h Activity Guidelines for Children and Adolescents

Caregivers reported the average hours children spent in sports, PA and active play in 30‐min increments over a 24‐h period on a typical weekday (0–7 h) and weekend day (0–12 h) (see Table S2), adapted from an existing survey (Ryan et al. 2024). The intraclass correlation coefficient (ICC) was used to assess agreement between the two versions of the self‐reported measures for time spent in MVPA and ST. The ICC estimates were calculated using a two‐way random effects model with absolute agreement and single measures. Caregivers who completed a second survey for the same child were used to conduct the test–retest reliability, excluding any responses that had been merged during the data management protocols. This survey showed excellent test–retest reliability (ICC = 0.905) in a subsample of caregivers (*n* = 16) (Cicchetti 1994).

Caregivers reported the average hours children spent using electronic screen devices in a sitting or lying position (excluding homework or educational activities) in 30‐min increments over a 24‐h period on a typical weekday (0–7 h) and weekend day (0–12 h). The question is also provided in Table S2, adapted from an existing survey (Ryan et al. 2024). The ICC was 0.734 for ST, suggesting good agreement (Cicchetti 1994). These questions were adopted from the existing movement behaviour surveys: WHO Global School–based student Health Survey (GSHS) (World Health Organization 2016); SUNRISE Study (Okely et al. 2021) and the Pacific Island–based Children's Healthy Living Program (Ryan et al. 2024; Wilken et al. 2013), with adjustments to reflect reporting of averages rather than daily compliance.

To determine if children met the Asia‐Pacific Integrated 24‐Hour guidelines, the weighted average of minutes spent in PA and ST per day was calculated using the formula: (weekday hours × 5) + (weekend hours × 2) / 7 days. Children were classified as meeting individual PA and ST recommendations if they met: (1) MVPA ≥ 60 min/day and (2) ST ≤ 2 h/day.

### 2.7 Sociodemographic Characteristics

**FIGURE 1:**
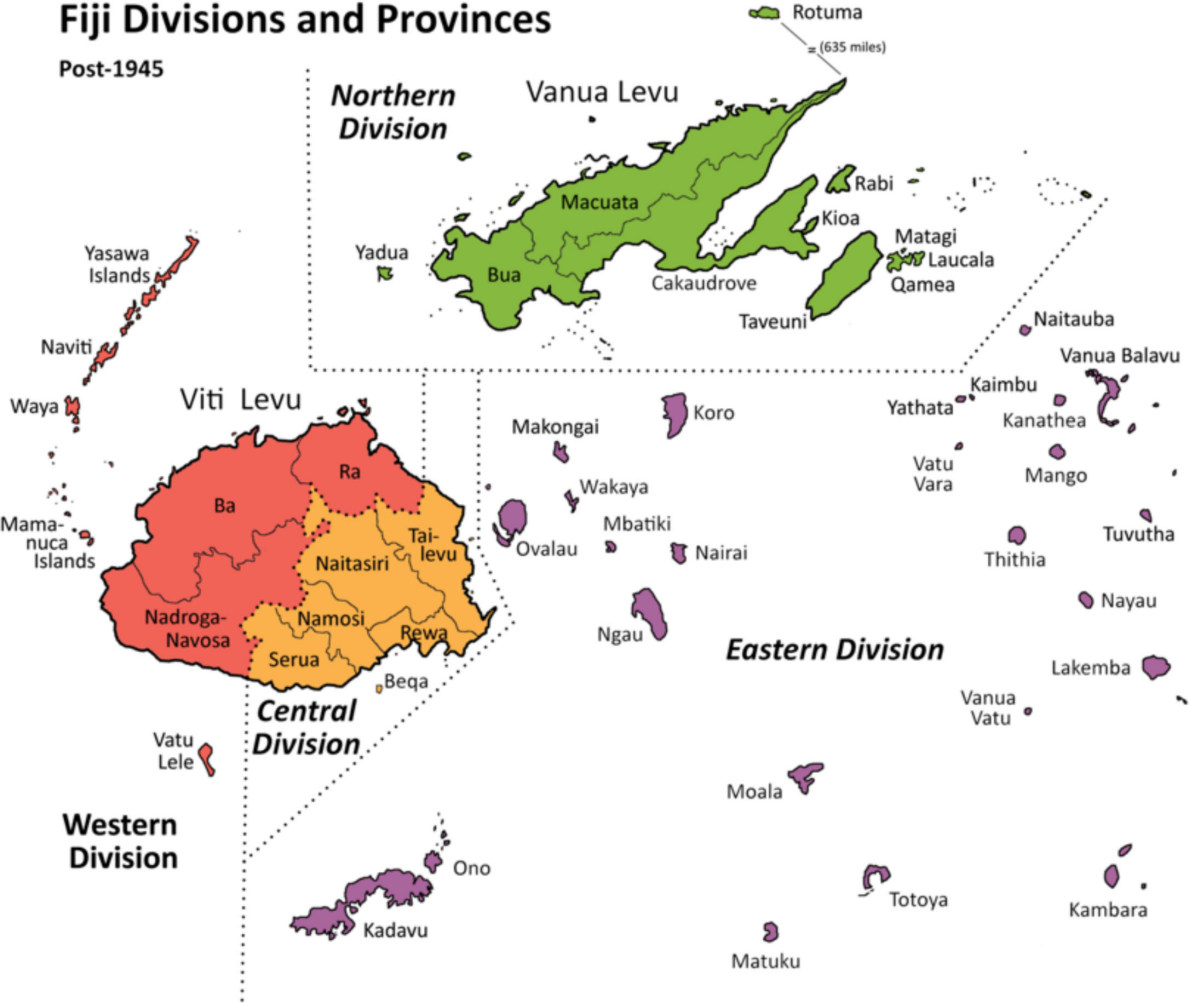
Map of Fijian divisions.

Caregivers provided information on the child's age (5–17 years), gender, predominant language spoken at home (Cantonese, English, Fijian Hindi, iTaukei/Fijian, Rotuman and other), caregivers' education level (no formal schooling, primary school, secondary or high school, vocational education, tertiary education or other) and division within Fiji (Central, Northern, Eastern and Western); see Figure 1. Caregivers selected the child's school based on the division's response, choosing from a list of primary or secondary schools within that geographic area. The region of each school was categorised as remote/very remote, rural or urban, based on data from the Fiji Ministry of Education.

### 2.8 Data Analysis

#### 2.8.1 Sample Size and Power Calculations

A sample size of 679 participants across 136 schools with an average of four caregiver/child dyads per school was calculated using an intraclass correlation coefficient (ICC) estimate of 0.11 (Parker et al. 2023). The sample size aimed to identify a four percentage point variance between male and female subjects in terms of meeting PA recommendations (Loo et al. 2022). This method is consistent with existing studies that use stratification sampling at a population level, such as the New South Wales Schools Physical Activity and Nutrition Survey (SPANS) (Okely et al. 2021; Booth and Dennev‐Wilson 2004) from Australia and the SUNRISE International Study (Okely et al. 2021). The calculation accounted for a significance threshold of 0.05 and a statistical power of 0.8.

#### 2.8.2 Data Management

For a survey attempt to be considered valid for inclusion in the analysis, caregivers had to complete the 12 demographic questions and two items on their child's time spent in MVPA. Duplicate entries from the same caregiver were identified by matching contact details and manually merged, prioritising the complete version based on the earlier commencement date. This merging only occurred if the caregiver had reached the guideline definitions section, to avoid potential carry‐over effects on subsequent knowledge and PA/ST duration items. Caregivers who completed a second survey for a different child were excluded due to possible cross‐influence on the PA and ST estimates.

#### 2.8.3 Statistical Analysis

Statistical analysis was conducted using *Jamovi* Software Version 2.3 (Sydney, Australia). Generalised linear mixed models (GLMMs), which account for random effects at the school, region and language spoken level, were used to analyse associations of meeting PA and ST recommendations, stratified by attitude, subjective norm and perceived behavioural control constructs. Language spoken and region (urban, rural, remote and very remote) have been identified as significant correlates within this sample and were explored to guide future social marketing initiatives (Ryan et al. 2025). These were presented as odds ratios (OR) and 95% confidence intervals (CI) for models meeting the PA and ST recommendations. Due to the simplified set of questions for younger children, results from children are divided by 5–8 and 9–17 years.

## 3 Results

### 3.1 Sample Description

**FIGURE 2:**
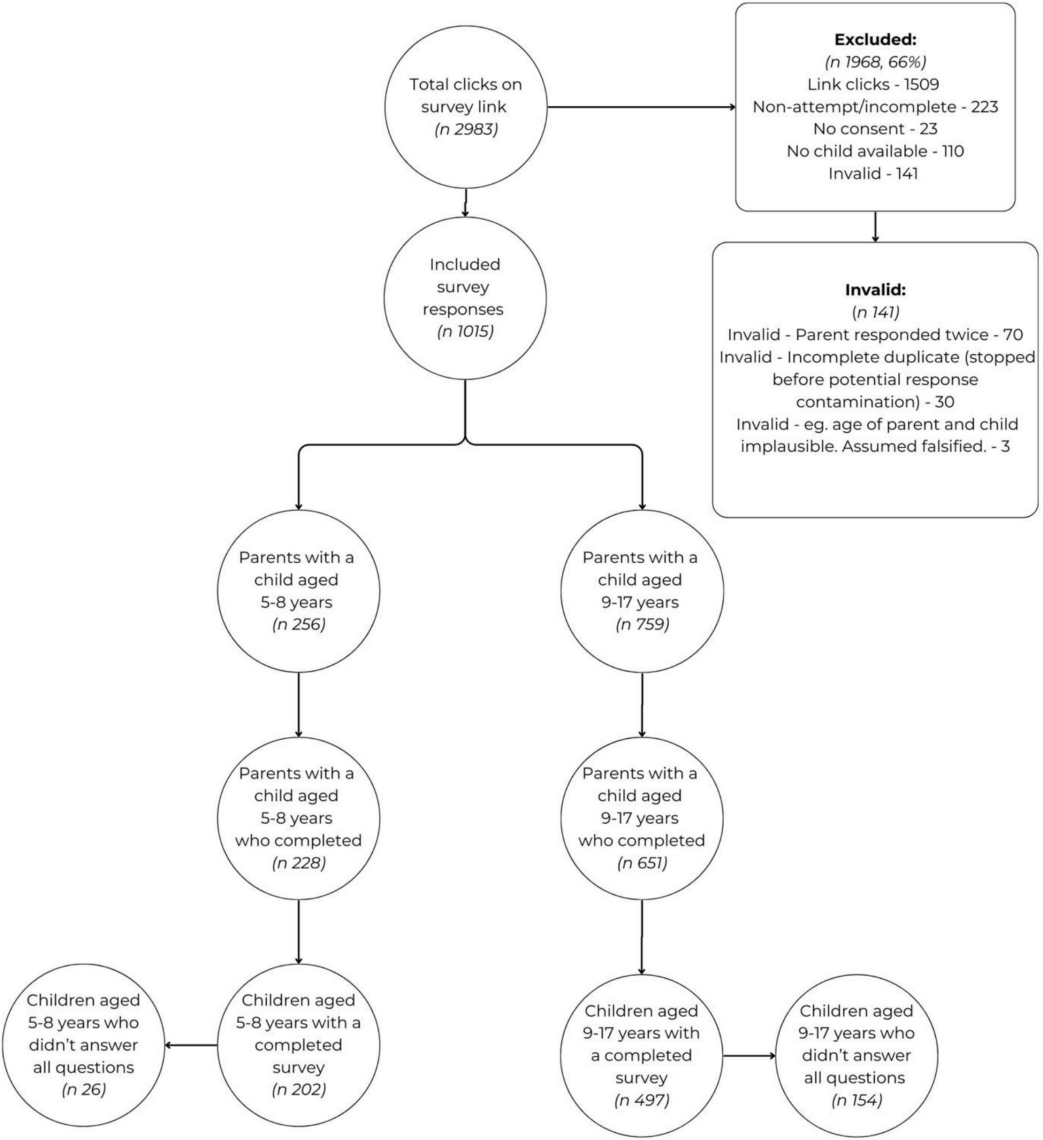
Flow chart.

The sample comprised 1015 caregivers (mean age 37.83 ± 8.43 years) and their children (mean age 11.39 ± 3.52 years), with 699 children completing the survey. Responses were obtained from 253 schools (174 primary and 79 secondary), with an average of four responses per school. Schools that received face‐to‐face visits had a higher response rate (*n* = 8). Among the caregivers, 80% were female, the relationship to the child was predominantly mothers (75%) and the majority (77%) of caregivers had attained a tertiary education degree. A flow chart of inclusion and exclusion criteria for respondents can be found in Figure 2.

Responses were collected nationally from Fiji's four divisions (see Figure 1 for a map), with 425 (42%) from the Central Division, 493 (49%) from the Western Division, 76 (7%) from the Northern Division and 21 (2%) from the Eastern Division. Most respondents (67%) lived in urban areas, whereas 15% were from rural areas and 18% from remote or very remote regions. The primary language spoken at home was iTaukei/Fijian (41%), followed by Fijian Hindi (36%) and English (22%).

### 3.2 Caregivers

Table 1 shows caregivers' attitudes, subjective norms and perceived behavioural control regarding PA and ST. Associations were tested between meeting and not meeting the respective guidelines.

**TABLE 1:** Caregiver attitudes, subjective norms and perceived behavioural control regarding physical activity and screen time.

Statement	Total responses (*n*)	Met screen time	Met physical activity
Attitude			
Caregivers' behavioural beliefs			
How important is it that your child …			
… is healthy?	599	—	0.913 (CI 0.565–1.476), *p* = 0.710
… is doing well at school?	959	—	1.197 (CI 0.767–1.868), *p* = 0.428
… has friends?	958	—	1.022 (CI 0.825–1.266), *p* = 0.843
… is happy?	959	0.904 (CI 0.669–1.220), *p* = 0.508	0.854 (CI 0.534–1.365), *p* = 0.509
… is not feeling stressed?	957	0.941 (CI 0.754–1.174), *p* = 0.592	—
… is not arguing with you?	958	0.991 (CI 0.840–1.169), *p* = 0.915	—
… is entertained and not feeling bored?	957	1.113 (CI 0.946–1.309), *p* = 0.198	—
Caregivers' evaluations of consequences			
If my child were to get 60 min every day of physical activity (that made them breathe heavily and huff and puff), it would …			
… help them be healthier.	926	—	0.947 (CI 0.720–1.246), *p* = 0.697
… help them concentrate and do better on their schoolwork or homework.	925	—	0.862 (CI 0.652–1.139), *p* = 0.295
… help my child form or keep friendships.	924	—	0.883 (CI 0.666–1.171), *p* = 0.389
… help make my child happier.	925	—	1.366 (CI 1.003–1.862), *p* = 0.048
If my child were to get no more than 2 h of recreational screen time (in a sitting or lying position) per day, it would …			
… help them be happier.	879	1.045 (CI 0.934–1.170), *p* = 0.443	—
… make them stressed.	880	1.114 (CI 0.955–1.300), *p* = 0.169	—
… cause arguments.	878	0.885 (CI 0.765–1.024), *p* = 0.102	—
… make them bored.	880	0.864 (CI 0.736–1.013), *p* = 0.071	—
Subjective norm			
Caregivers' normative beliefs			
… thinks it is important for my child to be physically active			
My partner …	911	—	1.286 (CI 0.994–1.665), *p* = 0.056
My family …	910	—	1.047 (CI 0.777–1.410), *p* = 0.764
My friends …	910	—	1.065 (CI 0.794–1.429), *p* = 0.674
My religious leaders …	910	—	0.775 (CI 0.579–1.037), *p* = 0.087
People on social media …	910	—	1.085 (CI 0.907–1.297), *p* = 0.373
… thinks it is important to restrict my child's screen time (in a sitting or lying position)			
My partner …	880	1.099 (CI 0.941–1.285), *p* = 0.232	—
My family …	880	0.995 (CI 0.820–1.206), *p* = 0.957	—
My friends …	880	0.996 (CI 0.829–1.196), *p* = 0.963	—
People on social media …	879	1.075 (CI 0.938–1.231), *p* = 0.300	—
Caregivers' motivation to comply			
Generally speaking, how much do you care what … thinks you should do?			
… your partner	958	1.082 (CI 0.917–1.277), *p* = 0.350	0.973 (CI 0.765–1.237), *p* = 0.822
… your family	957	1.127 (CI 0.955–1.330), *p* = 0.156	1.032 (CI 0.812–1.312), *p* = 0.794
… your friends	957	1.015 (CI 0.897–1.148), *p* = 0.815	0.986 (CI 0.822–1.183), *p* = 0.880
… your religious leaders	956	—	1.055 (CI 0.863–1.290), *p* = 0.600
… people on social media	956	0.968 (CI 0.876–1.069), *p* = 0.523	0.967 (CI 0.837–1.118), *p* = 0.652
Perceived behavioural control			
Caregivers' control beliefs			
Generally speaking, do you …			
… feel that you have enough money to do the things you want to do?	955	—	0.980 (CI 0.772–1.244), *p* = 0.869
… have access to transport to get where you need to go?	955	—	0.828 (CI 0.659–1.040), *p* = 0.104
… feel like you have enough time to do everything you want to do?	955	1.018 (CI 0.879–1.180), *p* = 0.810	1.070 (CI 0.846–1.352), *p* = 0.574
… feel that your neighbourhood is safe enough that you do not worry for your child's safety?	955	—	1.115 (CI 0.940–1.322), *p* = 0.213
… feel confident in your ability to play sport and be physically active with your child?	956	—	1.099 (CI 0.872–1.384), *p* = 0.423
… feel like screen time restrictions help your child spend less time on screens?	955	1.258 (CI 1.052–1.505), *p* = 0.012	—
… feel you have the ability to set screen time rules and restrictions for your child?	956	1.507 (CI 1.252–1.816), *p* ≤ 0.001	—
Caregivers' control factor salience			
Does … make it easier or more difficult to help your child get 60 min of physical activity per day?			
… having money available …	909	—	0.767 (CI 0.572–1.029), *p* = 0.077
… having access to transport …	909	—	1.225 (CI 0.932–1.610), *p* = 0.147
… having spare time …	910	—	0.949 (CI 0.693–1.298), *p* = 0.742
… feeling that your neighbourhood is safe …	910	—	1.073 (CI 0.827–1.391), *p* = 0.597
… your level of confidence or ability in playing sports or being physically active …	911	—	1.276 (CI 0.958–1.698), *p* = 0.095
Does … make it easier or more difficult for your child to spend less time on screens (sitting or lying down)?			
… having spare time …	880	1.136 (CI 0.953–1.355), *p* = 0.154	—
… using screen restrictions or rules …	875	1.290 (CI 1.055–1.577), *p* = 0.013	—
… your child's acceptance of screen time restrictions or rules …	879	0.988 (CI 0.813–1.202), *p* = 0.908	—

#### 3.2.1 Attitudes: Behavioural Beliefs

No statistically significant associations were found for any of the caregiver behavioural beliefs.

#### 3.2.2 Attitudes: Evaluation of Consequences

When caregivers believed that meeting PA recommendations would make their child happier, their children were 1.4 times more likely to meet the PA recommendation, compared to not meeting it (OR = 1.366, CI 1.003–1.862).

#### 3.2.3 Subjective Norm: Normative Beliefs

No statistically significant associations were found for any of the normative caregiver beliefs.

#### 3.2.4 Subjective Norm: Motivation to Comply

Caregivers varied in the extent to which they were motivated to comply with different stakeholder groups. Caregivers were most commonly motivated to comply with their family members (87%) and partners (85%). Caregivers were less motivated to comply with what religious leaders (70%) or friends (55%) thought they should do. Caregivers were least likely to care about what people on social media thought they should do (56%). Further details on caregivers' motivation to comply are reported in Table S1 and Tables S4 and S5 by language spoken and region.

No statistically significant associations were found for any of the caregivers' motivation to comply variables.

#### 3.2.5 Perceived Behavioural Control: Control Beliefs

When caregivers believed that ST restrictions would help their child spend less time on screens, their children were 1.3 times more likely to meet the ST recommendation, compared to not meeting it (OR = 1.258, CI 1.052–1.505). Additionally, as caregivers' confidence in setting ST restrictions increased, children were 1.5 times more likely to meet the ST recommendation, compared to not meeting it (OR = 1.507, CI 1.252–1.816).

#### 3.2.6 Perceived Behavioural Control: Control Factor Salience

When caregivers believed that using screen rules and restrictions would make it easier for their child to meet ST recommendations, their children were 1.3 times more likely to meet the ST recommendation, compared to not meeting it (OR = 1.290, CI 1.055–1.577).

#### 3.2.7 Trust in Institutions/Groups to Provide Health Information

Caregivers reported the highest level of trust in the Ministry of Education, teachers and schools and PA and sports programmes to provide health information about their child (all at 91%). Caregivers reported having the lowest level of trust in community leaders (65%) and social media (48%) (see Table 2). Religious institutions were trusted by 71% of caregivers, with higher levels among iTaukei/Fijian (77%) and lower levels among Fijian Hindi and English‐speaking caregivers (66%) (see Table S6). There was little variation across rural, remote and urban levels in terms of who caregivers trusted to provide health‐related information (see Table S7).

**TABLE 2:** Caregivers trust in institutions/groups to provide health information.

Organisation/agencies	Total responses (*n*)	Disagree *n* (%)	Neither agree nor disagree *n* (%)	Agree *n* (%)
Ministry of health	987	34 (3)	71 (7)	882 (90)
Ministry of education	987	25 (2)	67 (7)	895 (91)
The ministry of youth & sports	984	39 (4)	128 (13)	817 (83)
Teachers and schools	987	20 (2)	73 (7)	894 (91)
Physical activity and sports programmes	986	18 (2)	73 (7)	895 (91)
Community leaders	985	116 (12)	226 (23)	643 (65)
Religious institutions	985	93 (9)	197 (20)	695 (71)
Social media (e.g., Facebook & Instagram)	985	232 (24)	276 (28)	477 (48)

### 3.3 Younger Children Aged 5–8 Years

Attitudes, subjective norms and perceived behavioural control to PA and ST in younger children aged 5–8 years are presented in Table 3. Associations were tested between meeting and not meeting the respective guidelines.

**TABLE 3:** Children aged 5–8 years: Attitudes, subjective norms and perceived behavioural control regarding physical activity and screen time.

Statement	Total responses (*n*)	Met screen time	Met physical activity
Attitude			
Children's (5–8) behavioural beliefs			
How important is it that you …			
… are doing well in school?	208	3.096 (CI 0.263–36.516), *p* = 0.369	2.068 (CI 0.078–55.102), *p* = 0.664
… are happy?	207	N/A	N/A
… are having fun?	207	2.755 (CI 0.221–34.309), *p* = 0.431	N/A
… are healthy and strong?	207	—	5.295 (CI 0.224–124.921), *p* = 0.301
… have friends?	207	1.372 (CI 0.444–4.247), *p* = 0.583	2.886 (CI 0.471–17.692), *p* = 0.252
Children's (5–8) evaluation of consequences			
If you were to get 60 min every day of playing or physical activity (that made you breathe heavily and huff and puff), it would help you …			
… do better on your schoolwork.	204	—	5.464 (CI 1.348–22.139), *p* = 0.017
… be happier.	204	—	16.529 (CI 1.227–222.665), *p* = 0.035
… have more fun.	204	—	0.078 (CI 0.004–1.436), *p* = 0.086
… be healthier and stronger.	204	—	0.031 (CI 0.001–0.802), *p* = 0.036
… to make or keep friendships.	204	—	2.064 (CI 0.402–10.595), *p* = 0.385
If you were to get 2 h or less of recreational screen time every day (in a sitting or lying position), it would help you …			
… to have more fun.	202	1.116 (CI 0.358–3.482), *p* = 0.850	—
… to do better in your schoolwork.	202	1.769 (CI 0.712–4.397), *p* = 0.219	—
… to be happier.	202	0.500 (CI 0.148–1.691), *p* = 0.265	—
… to make or keep friendships.	202	1.763 (CI 0.750–4.142), *p* = 0.193	—
Subjective norm			
Children's (5–8) normative beliefs			
… think it is important to be physically active			
My family …	204	—	0.680 (CI 0.035–13.376), *p* = 0.800
My friends …	204	—	2.347 (CI 0.300–18.368), *p* = 0.416
My religious leaders …	204	—	1.177 (CI 0.201–6.891), *p* = 0.856
Sports champions I admire …	204	—	2.530 (CI 0.376–17.007), *p* = 0.340
My teachers …	204		N/A
Think it is important to restrict my screen time (in a sitting or lying position)			
My family …	201	0.456 (CI 0.118–1.758), *p* = 0.254	—
My friends …	202	1.039 (CI 0.499–2.162), *p* = 0.919	—
My religious leaders …	202	1.987 (CI 0.833–4.740), *p* = 0.122	—
My teachers …	202	2.639 (CI 0.694–10.031), *p* = 0.154	—
Children's (5–8) motivation to comply			
How much do you care what … think you should do?			
… your family	207	1.213 (CI 0.180–8.168), *p* = 0.843	1.116 (CI 0.072–17.359), *p* = 0.938
… your friends	206	0.545 (CI 0.240–1.238), *p* = 0.147	1.380 (CI 0.404–4.712), *p* = 0.607
… your religious leaders	206	5.393 (CI 2.423–12.002), *p* ≤ 0.001	2.123 (CI 0.532–8.476), *p* = 0.287
… spots champions …	206	—	0.532 (CI 0.122–2.322), *p* = 0.401
… your teachers …	206	N/A	N/A
Perceived behavioural control			
Children's (5–8) control beliefs			
Generally speaking, do you …			
… feel like you have somewhere near your home to play when you want to be active?	206	—	3.539 (CI 0.702–17.839), *p* = 0.126
… feel the weather stops you from being active when you want to be?	206	—	1.795 (CI 0.671–4.804), *p* = 0.244
… feel you have enough equipment or toys available when you want to be active?	206	—	1.958 (CI 0.725–5.289), *p* = 0.185
… feel like screen time rules and restrictions stop you from using screens when you want to?	206	0.751 (CI 0.397–1.421), *p* = 0.379	—
… feel like you have access to screens or tablets when you want to use them?	206	0.469 (CI 0.224–0.984), *p* = 0.045	—
Children's (5–8) control factor salience			
Does … make it easier or more difficult to for you to get 60 min of physical activity per day?			
… having somewhere to play near my home …	204	—	1.891 (CI 0.302–11.825), *p* = 0.496
… the weather …	204	—	1.160 (CI 0.442–3.044), *p* = 0.763
… the toys or equipment available to you …	204	—	3.062 (CI 0.829–11.303), *p* = 0.093
Does … make it easier or more difficult for you to spend less time on screens (sitting or lying down)?			
… having screen time restrictions or rules …	202	1.443 (CI 0.648–3.216), *p* = 0.370	—
… having to share screens or tablets …	202	1.019 (CI 0.515–2.018), *p* = 0.957	—

#### 3.3.1 Attitudes: Behavioural Beliefs

No statistically significant associations were found for any of the younger children aged 5–8 years' behavioural beliefs.

#### 3.3.2 Attitudes: Evaluation of Consequences

For younger children aged 5–8 years, believing that getting 60 min MVPA would help them do better with schoolwork was associated with a 5.5 times higher likelihood of meeting PA recommendation, compared to not meeting it (OR = 5.464, 95% CI 1.348–22.139). Children who believed that 60 min of MVPA would help them be happier were 16.5 times more likely to meet PA recommendation, compared to not meeting it (OR = 16.529, 95% CI 1.227–222.665). Interestingly, children who believed MVPA would help them be healthy and stronger were 0.03 times less likely to meet the PA recommendation, compared to not meeting it (OR = 0.031, 95% CI 0.001–0.802).

#### 3.3.3 Subjective Norm: Normative Beliefs

No statistically significant associations were found for any of the younger children aged 5–8 years' normative beliefs.

#### 3.3.4 Subjective Norm: Motivation to Comply

Younger children aged 5–8 years are highly motivated to comply with family members (96%) and teachers (98%). Friends and sports champions influenced 76% of children, whereas religious leaders motivated 78% of children. Only 2% of children do not care about complying with their teachers. Further details on children aged 5–8 years' motivation to comply are reported in Table S8 and Tables S9 and S10 by language spoken and region.

When younger children aged 5–8 years were motivated to comply with their religious leaders, they were 5.4 times more likely to meet the ST recommendation, compared to not meeting it (OR = 5.393, CI 2.423–12.002).

#### 3.3.5 Perceived Behavioural Control: Control Beliefs

When younger children aged 5–8 years believed they had easy access to screens or tablets whenever they wanted, they were 0.5 times less likely to meet the ST recommendation, compared to not meeting it (OR = 0.469, CI 0.224–0.984).

#### 3.3.6 Perceived Behavioural Control: Control Factor Salience

No statistically significant associations were found for any of the younger children aged 5–8 years' control factor salience variables.

### 3.4 Older Children/Adolescents Aged 9–17 Years

Table 4 presents the data on attitudes, subjective norms and perceived behavioural control of PA and ST for older children/adolescents aged 9–17 years. Associations were tested between meeting and not meeting the respective guidelines.

**TABLE 4:** Older children/adolescents aged 9–17 years: Attitudes, subjective norms and perceived behavioural control regarding physical activity and screen time.

Statement	Total responses (*n*)	Met screen time	Met physical activity
Attitude			
Older children/adolescents' (9–17) behavioural beliefs			
How important is it that you …			
… are doing well in school?	523	0.188 (CI 0.020–1.791), *p* = 0.146	0.572 (CI 0.040–8.112), *p* = 0.680
… are happy?	524	0.421 (CI 0.050–3.543), *p* = 0.426	N/A
… are having fun?	524	2.931 (CI 0.893–9.621), *p* = 0.076	1.671 (CI 0.283–9.886), *p* = 0.571
… are healthy and strong?	523	—	N/A
… have friends?	523	0.817 (CI 0.413–1.614), *p* = 0.561	0.368 (CI 0.113–1.197), *p* = 0.097
… are not feeling bored?	523	1.220 (CI 0.665–2.236), *p* = 0.521	0.791 (CI 0.318–1.965), *p* = 0.613
… are not feeling embarrassed?	523	—	2.809 (CI 1.008–7.830), *p* = 0.048
… are not feeling anger or frustration?	523	0.869 (CI 0.476–1.586), *p* = 0.646	0.867 (CI 0.287–2.621), *p* = 0.801
… are not being bullied?	523	—	0.530 (CI 0.142–1.985), *p* = 0.346
Older children/adolescents' (9–17) evaluation of consequences			
If you were to get 60 min every day of playing or physical activity (that made you breathe heavily and huff and puff), it would help you …		—	
… do better on your schoolwork.	505	—	1.617 (CI 0.778–3.361), *p* = 0.198
… be happier.	505	—	0.495 (CI 0.161–1.515), *p* = 0.218
… have more fun.	505	—	2.114 (CI 0.695–6.426), *p* = 0.187
… be healthier and stronger.	505	—	0.810 (CI 0.153–4.273), *p* = 0.803
… to make or keep friendships.	505	—	1.289 (CI 0.576–2.882), *p* = 0.536
… to be entertained and not feel bored.	504	—	0.375 (CI 0.106–1.326), *p* = 0.128
… to feel less embarrassed.	505	—	1.318 (CI 0.626–2.776), *p* = 0.467
… with feelings of anger or frustration.	505	—	0.800 (CI 0.378–1.695), *p* = 0.561
… with bullying.	505		0.897 (CI 0.464–1.736), *p* = 0.747
If you were to get 2 h or less of recreational screen time every day (in a sitting or lying position), it would help you …			
… to do better in your schoolwork.	493	0.493 (CI 0.287–0.849), *p* = 0.011	—
… to be happier.	493	1.168 (CI 0.667–2.047), *p* = 0.587	—
… to have more fun.	493	1.943 (CI 1.047–3.606), *p* = 0.035	—
… to make or keep friendships.	492	0.704 (CI 0.394–1.258), *p* = 0.236	—
… with boredom.	492	0.961 (CI 0.559–1.651), *p* = 0.885	—
… with feelings of anger or frustration.	493	0.866 (CI 0.508–1.475), *p* = 0.596	—
Subjective norm			
Older children/adolescents' (9–17) normative beliefs			
… think it is important to be physically active			
My family …	503	—	0.414 (CI 0.046–3.715), *p* = 0.431
My friends …	505	—	0.800 (CI 0.303–2.115), *p* = 0.653
My religious leaders …	505	—	1.227 (CI 0.523–2.877), *p* = 0.638
Sports champions I admire …	504	—	1.632 (CI 0.652–4.089), *p* = 0.296
My teachers …	505	—	0.935 (CI 0.243–3.607), *p* = 0.923
People on social media …	504	—	1.278 (CI 0.695–2.351), *p* = 0.430
… think it is important to restrict my screen time (in a sitting or lying position)			
My family …	493	1.361 (CI 0.535–3.467), *p* = 0.518	—
My friends …	493	1.557 (CI 0.897–2.700), *p* = 0.115	—
My religious leaders …	493	0.784 (CI 0.400–1.538), *p* = 0.479	—
My teachers …	493	0.603 (CI 0.232–1.567), *p* = 0.299	—
People on social media …	492	0.861 (CI 0.519–1.430), *p* = 0.564	—
Older children/adolescents' (9–17) motivation to comply			
How much do you care what … think you should do?			
… your family	523	0.841 (CI 0.293–2.414), *p* = 0.747	1.497 (CI 0.437–5.132), *p* = 0.521
… your friends	523	1.052 (CI 0.656–1.686), *p* = 0.835	1.381 (CI 0.755–2.525), *p* = 0.294
… your religious leaders	522	1.079 (CI 0.634–1.835), *p* = 0.780	0.696 (CI 0.330–1.469), *p* = 0.341
… spots champions …	522	2.528 (CI 0.669–9.558), *p* = 0.172	1.169 (CI 0.617–2.213), *p* = 0.632
… your teachers …	522	0.964 (CI 0.617–1.506), *p* = 0.872	1.138 (CI 0.226–5.746), *p* = 0.875
… people on social media	522	0.841 (CI 0.293–2.414), *p* = 0.747	1.526 (CI 0.802–2.903), *p* = 0.198
Perceived behavioural control			
Older children/adolescents's (9–17) control beliefs			
Generally speaking, do you …			
… feel like you have somewhere near your home to play when you want to be active?	522	—	1.325 (CI 0.526–3.340), *p* = 0.550
… feel the weather stops you from being active when you want to be?	522	—	0.672 (CI 0.388–1.165), *p* = 0.157
… feel you have enough equipment or toys available when you want to be active?	521	—	0.953 (CI 0.545–1.668), *p* = 0.867
… feel like screen time rules and restrictions stop you from using screens when you want to?	521	0.939 (CI 0.603–1.463), *p* = 0.782	—
… feel like you have access to screens or tablets when you want to use them?	520	0.510 (CI 0.324–0.802), *p* = 0.004	—
… feel like you have enough time to do everything you want to do?	520	—	0.979 (CI 0.551–1.739), *p* = 0.943
… feel like you have the confidence or ability to participate in physical activity?	521	—	2.656 (CI 0.983–7.175), *p* = 0.054
… feel like you could walk to school if you wanted to?	521	—	1.131 (CI 0.639–2.003), *p* = 0.672
Older children/adolescents' (9–17) control factor salience			
Does … make it easier or more difficult to for you to get 60 min of physical activity per day?			
… having somewhere to play near my home …	504	—	0.909 (CI 0.223–3.707), *p* = 0.894
… the weather …	504	—	1.276 (CI 0.741–2.196), *p* = 0.380
… the toys or equipment available to you …	504	—	0.634 (CI 0.266–1.508), *p* = 0.303
… the amount of spare time you have …	503	—	2.865 (CI 1.116–7.354), *p* = 0.029
… walking to school …	503	—	0.635 (CI 0.298–1.353), *p* = 0.239
… having confidence and the ability to participate …	503		1.263 (CI 0.341–4.673), *p* = 0.726
Does … make it easier or more difficult for you to spend less time on screens (sitting or lying down)?			
… having screen time restrictions or rules …	493	1.177 (CI 0.687–2.016), *p* = 0.552	—
… having to share screens or tablets …	493	1.042 (CI 0.628–1.729), *p* = 0.874	—

#### 3.4.1 Attitudes: Behavioural Beliefs

When older children/adolescents aged 9–17 years believed it was important to not feel embarrassed, they were 2.8 times more likely to meet the PA recommendation, compared to not meeting it (OR = 2.809, CI 1.008–7.830).

#### 3.4.2 Attitudes: Evaluation of Consequences

When older children/adolescents aged 9–17 years believed that meeting ST recommendations (no more than 2 h per day) would help them do better at homework, they were 0.5 times less likely to meet ST recommendation, compared to not meeting it (OR = 0.493, CI 0.287–0.849). However, when these children believed that meeting ST recommendations would help them have more fun, they were 1.9 times more likely to meet the ST recommendation, compared to not meeting it (OR = 1.943, CI 1.047–3.606).

#### 3.4.3 Subjective Norm: Normative Beliefs

No statistically significant associations were found for any of the older children/adolescents aged 9–17 years' normative beliefs.

#### 3.4.4 Subjective Norm: Motivation to Comply

Older children/adolescents aged 9–17 years showed high motivation to comply with family members (96%) and teachers (98%). Friends influenced 71% of children, whereas sports champions and religious leaders motivated 76% and 83%, respectively. Only 2% of children did not care about complying with their teachers. Further details on older children/adolescents aged 9–17 years' motivation to comply are reported in Table S11 and Tables S12 and S13 by language spoken and region.

No statistically significant associations were found for any of the older children/adolescents aged 9–17 years' motivation to comply variables.

#### 3.4.5 Perceived Behavioural Control: Control Beliefs

When older children/adolescents aged 9–17 years felt they had easy access to screens or tablets whenever they wanted, they were 0.5 times less likely to meet the ST recommendation, compared to not meeting it (OR = 0.510, CI 0.324–0.802).

#### 3.4.6 Perceived Behavioural Control: Control Factor Salience

When older children/adolescents aged 9–17 years felt that having more spare time made it easier to meet 60 min of MVPA per day, they were 2.9 times more likely to meet the PA recommendation, compared to not meeting it (OR = 2.865, CI 1.116–7.354).

## 4 Discussion

We examined Fijian caregivers' and children aged 5–17 years' attitudes, subjective norms and perceived behavioural control for PA and ST and how these factors are associated with the likelihood of meeting the recommendations for PA and ST. In this discussion, the findings are organised thematically, grouping related results across age groups and caregiver data, rather than strictly following the order presented in the results tables. This is to provide a more cohesive analysis of the patterns observed across different associated factors.

### 4.1 Factors Associated With Meeting the Guidelines

#### 4.1.1 PA and Happiness

Caregivers and children aged 5–9 years who believe that participating in PA makes their child/them happier were more likely to meet the PA recommendation. This is supported by evidence showing children who participate in PA have higher levels of happiness (van Woudenberg et al. 2020). The significant association between this belief and meeting PA recommendations highlights the need to promote the mental health benefits of PA, particularly in an era when mental health is at the forefront of the health agenda. Although this association was statistically significant, the wide confidence interval means results should be applied with caution. The wide interval is likely attributed to the high level of agreement (~90% of children 5–9 years) reported believing PA contributes to happiness.

#### 4.1.2 ST Restrictions

Caregivers who believe that ST restrictions are effective and who feel capable of implementing ST restrictions were more likely to have children who meet the ST recommendations. Restrictions are effective in reducing children's ST; however, parental role modelling is also an important aspect (Schoeppe et al. 2016). Our finding that children aged 5–17 years who believed they had easy access to screens were less likely to meet ST recommendations further reinforces the importance of caregiver‐implemented restrictions. Future interventions should focus on supporting and empowering caregivers in implementing and adhering to ST recommendations, possibly through training in effective communication styles (Bjelland et al. 2015). However, this would need to be investigated at the country level to ensure context and the needs are clearly understood.

#### 4.1.3 Academic Performance

Younger children aged 5–8 years who believe that participating in PA would improve their schoolwork were more likely to meet the PA recommendation. This aligns with research showing that PA enhances academic performance (James et al. 2023). Given the high value placed on academic performance in some Fijian cultures (Otsuka 2006), this finding suggests a potential avenue for promoting PA through its academic benefits. Interestingly, 9‐17‐year‐olds who believed less ST would help their schoolwork were less likely to meet ST recommendations. It may be that these adolescents are more focused on immediate gratification and socialising with friends on social media, which aligns with evidence showing that adolescents' brains are particularly sensitive to rewarding stimuli, including online interactions such as Likes, driving a preference for short‐term rewards over long‐term benefits (Marciano et al. 2021).

#### 4.1.4 PA and Health

Younger children aged 5–8 years who believe PA makes them healthier and stronger were less likely to meet the PA recommendations. This finding seems counterintuitive; however, this group of children may place more value on the emotional benefit of being happy than the more rational benefit of being healthy. This may be explained by the natural tendency for younger children to prioritise instant gratification over delayed benefits such as long‐term health (McCormack et al. 2024). Alternatively, it may indicate a misalignment between children's beliefs and behaviour. Fijian children aged 5–8 years may understand the health benefits of PA, but this knowledge may not translate into actual behaviour change. There could be several explanations for this. For example, the authors hypothesise there may be a misunderstanding of the term PA, and younger children may associate this with structured sports rather than active play. There may also be barriers such as time and resources that have not been captured within this study that warrant further investigation on the misalignment or the potential of social desirability bias in responses. Future research should explore this discrepancy, possibly using qualitative methods to gain deeper insights into children's understanding and motivations.

#### 4.1.5 Trusted Leaders and Institutions

Younger children aged 5–8 years who believe it is important to follow the guidance of their religious leaders were more likely to meet the ST recommendation. This highlights the potential for using religious leaders as messengers to promote the importance of meeting ST recommendations, particularly for younger children. Given the high importance of religion in Fijian society across ethnicities (Fiji Bureau of Statistics 2017), religious leaders could play an important role in health promotion. It is important to note that this approach may be less effective for caregivers and older children, emphasising the need for age‐specific strategies. Seventy percent of caregivers, 78% of younger children aged 5–8 years and 83% of 9–17 year olds stated they ‘care what their religious leaders think they should do’. Notably, 98% of children care what their teachers think they should do, reinforcing their significant influence. Although 71% of caregivers trust religious leaders to provide health information regarding their child's PA and ST, 90%–91% trust the Ministry of Health and Education. This indicates that although high trust is placed on community leaders, they may need support from the Ministry of Health to promote formalised messages. This suggests a potential strategy of collaborating with various trusted figures and health authorities to deliver consistent, trusted health messages.

#### 4.1.6 Embarrassment

Older children/adolescents aged 9–17 years who believe it is important to not feel embarrassed were more likely to meet the PA recommendations. It is important to note that this was asked generally before any questions relating to PA and ST commenced, highlighting the importance of emotional motivators in this age group. This finding aligns with the idea that children are driven by emotional benefits, such as social acceptance, rather than rational motivators such as health or academic success. In addition, children who participate in PA are more likely to experience positive self‐esteem and feelings of achievement (Peralta et al. 2022), which may reduce feelings of embarrassment. Given the associations between children's early experiences with PA and their likelihood of remaining active as adults (Costa et al. 2021), addressing feelings of embarrassment could be key to promoting long‐term PA engagement. Interventions that focus on creating supportive, nonjudgmental environments for PA could be particularly effective, especially for girls where these barriers are evident globally (Guthold et al. 2022).

#### 4.1.7 Fun

Older children/adolescents aged 9–17 who believed that having less ST would lead to more fun were more likely to meet the ST recommendations. This could indicate that older children are using screens out of boredom, suggesting that creating more opportunities for fun through PA might reduce ST and increase PA levels. Prior research has found that parents of Pacific Islands' children and adolescents report that fun and engaging physical activities are the most popular motivator for children participating in regular physical activities (Peralta et al. 2022). These findings reinforce the importance of emotional motivators in children as means to promote healthy movement behaviours.

#### 4.1.8 PA and Spare Time

Older children/adolescents aged 9–17 years who believe having more spare time facilitates meeting PA recommendations were more likely to meet the recommendations. Time, lack of motivation and discomfort were found to be the top three barriers to engaging in PA (Peralta et al. 2022). Further investigation into the specific time constraints older children face in Fiji (e.g., increased travel time to school, higher academic and family responsibilities) is needed to address this barrier effectively. However, it does highlight the need for interventions to support children and families in integrating PA into their daily routines, rather than viewing it as an additional time commitment.

### 4.2 Limitations and Future Research

Limitations include parent‐reported PA and ST measures, which may be subject to social desirability bias or recall errors. Where possible, future studies could include device‐based measures, such as accelerometers for PA. Although efforts were made to ensure a diverse sample, the study may not fully represent all demographic and geographic areas of Fiji. Our sample showed an overrepresentation (77% compared to 23%) of individuals with tertiary education (Fiji Bureau of Statistics 2022) likely due to the online survey method, which required internet access. This led to a lower response rate from the Eastern Division, where internet connections are limited (Fiji Bureau of Statistics 2022). The low response rate reflects the challenges of conducting surveys, specifically internet‐based surveys across Fiji's 332 islands, where digital access varies considerably. For some analyses, the small number of children in the comparison group resulted in imprecise estimates and wide confidence intervals. Qualitative studies could provide deeper insights into the reasons behind some of the counterintuitive findings, such as the emotional motivations of children and adolescents over the rational motivators. In addition, intervention studies testing the effectiveness of the proposed social marketing strategies would be valuable in translating these findings into practical public health initiatives.

## 5 Conclusion

This study explored the factors associated with caregivers and aged 5–17 years attitudes, subjective norms and perceived behavioural control towards meeting PA and ST recommendations. Findings reveal several factors that are associated with children meeting recommendations, including the association between PA and happiness, the importance of caregiver‐implemented ST restrictions and the potential influence of religious leaders and teachers. Our research highlights the importance of tailored strategies to leverage teachers and religious leaders for younger children, and ministries and schools for caregivers, ensuring consistent health messaging across trusted channels. The implications for public health policy and practice in Fiji include the need for interventions that address not only individual attitudes and beliefs but also social norms and environmental factors that either hinder or facilitate performance of the desired behaviours. Incorporating this research to build supportive environments that encourage PA and manage ST will contribute to the future health of all Fijians.

## Author Contributions

**Sarah T. Ryan:** conceptualisation, methodology, formal analysis, writing – original draft. **Anthony D. Okely:** conceptualisation, methodology, supervision, writing – review and editing. **Rebecca M. Stanley:** writing – review and editing, supervision. **Gade Waqa:** writing – review and editing, supervision. **Melanie Randle:** conceptualisation, methodology, writing – review and editing, supervision.

## Funding

This work was supported by the National Health and Medical Research Council (GNT 1176858).

## Conflicts of Interest

The authors declare no conflicts of interest.

## Supporting information


**Table S1:** Checklist for Reporting Results of Internet E‐Surveys (CHERRIES).
**Table S2:** Methodology Details.
**Table S3:** Caregivers' motivation to comply with certain people.
**Table S4:** Caregivers' motivation to comply by language group (iTaukei/Fijian, Fijian Hindi, English).
**Table S5:** Caregivers' motivation to comply by region (urban, rural, remote & very remote).
**Table S6:** Caregivers trust in institutions/groups to provide health information by Language Group (iTaukei/Fijian, Fijian Hindi, English).
**Table S7:** Caregivers trust in institutions/groups to provide health information by Region (Urban, Rural, Remote & Very Remote).
**Table S8:** Children aged 5–8 years' motivation to comply with certain people.
**Table S9:** Children 5–8 years motivation to comply by language group (iTaukei/Fijian, Fijian Hindi, English).
**Table S10:** Children 5–8 years motivation to comply by region (urban, rural, remote & very remote).
**Table S11:** Children aged 9–17 years' motivation to comply with certain people.
**Table S12:** Children 9–17 years motivation to comply by language group (iTaukei/Fijian, Fijian Hindi, English).
**Table S13:** Children 9–17 years motivation to comply by region (urban, rural, remote & very remote).

## Data Availability

The data that support the findings of this study are available on request from the corresponding author. The data are not publicly available due to privacy or ethical restrictions.
