# Supplementary material for: Attitudes, Norms, and Control: What Is Shaping Fijian Children's Physical Activity and Screen Time Behaviours?
Source: Child Care Health Dev. 2026 Mar 18;52(2):e70256. doi: 10.1111/cch.70256 (PMC12999357; doi:10.1111/cch.70256)
Supplement: Supplementary file 1 — Table S1: Checklist for Reporting Results of Internet E‐Surveys (CHERRIES). Table S2: Methodology Details. Table S3: Caregivers' motivation to comply with certain people. Table S4: Caregivers' motivation to comply by language group (iTaukei/Fijian, Fijian Hindi, English). Table S5: Caregivers' motivation to comply by region (urban, rural, remote & very remote). Table S6: Caregivers trust in institutions/groups to provide health information by Language Group (iTaukei/Fijian, Fijian Hindi, English). Table S7: Caregivers trust in institutions/groups to provide health information by Region (Urban, Rural, Remote & Very Remote). Table S8: Children aged 5–8 years' motivation to comply with certain people. Table S9: Children 5–8 years motivation to comply by language group (iTaukei/Fijian, Fijian Hindi, English). Table S10: Children 5–8 years motivation to comply by region (urban, rural, remote & very remote). Table S11: Children aged 9–17 years' motivation to comply with certain people. Table S12: Children 9–17 years motivation to comply by language group (iTaukei/Fijian, Fijian Hindi, English). Table S13: Children 9–17 years motivation to comply by region (urban, rural, remote & very remote). [file CCH-52-e70256-s001.docx]

Supplementary File:

Table A1: Checklist for Reporting Results of Internet E-Surveys (CHERRIES)

| Checklist Category | Checklist Item | Explanation |
| --- | --- | --- |
| Design | Describe survey design | Our open survey utilised a broad, multi-modal recruitment approach, to obtain a national convenience sample of caregivers and one of their children aged 5-17 years living in Fiji. |
| IRB (Institutional Review Board) approval and informed consent process | IRB approval | The study was approved by the University of Wollongong Social Sciences Human Research Ethics Committee (#2019/199), the Fiji National University College Human Health Research Ethics Committee (#175.20), and the Fiji Ministry of Education, Heritage and Arts (#RA 5/22). |
|  | Informed consent | Caregivers provided informed consent for their own participation as well as for one of their children to complete the child component of the survey. Upon commencing the caregiver's portion, simplified survey information was provided to obtain tacit assent from the child participant. |
|  | Data protection | Data were collected and managed using REDCap electronic data capture tools hosted at The University of Wollongong, ensuring compliance with the General Data Protection Act (GDPA) for secure data transfer and storage in Australia. |
| Development and pre-testing | Development and testing | The survey questions were developed with experts in physical activity and social marketing to ensure alignment with the theory of planned behaviour and evidence-based methods of collecting physical activity data. Local contacts and co-authors, GW and MK, reviewed the survey for cultural appropriateness and suitability to the local context. The research team, along with Australian children and caregivers, tested the technical functionality. Beta testing was then conducted with caregivers and children in Fiji. Additionally, survey data were tested in a small population in French Polynesia to assess suitability across the wider Pacific Region. French Polynesia was chosen as a test site due to its cultural and geographical diversity, which shares similarities with many other Pacific Island Countries and Nations. While it has its unique characteristics, it represents a mix of Polynesian culture and French influence, providing insights into how the survey might perform in various Pacific contexts. This version was translated into French and delivered as an interview, allowing researchers to evaluate respondents' comprehension across language barriers and cultural nuances. |
| Recruitment process and description of the sample having access to the questionnaire | Open survey versus closed survey | Open survey available to all caregivers of children aged 5-17 in Fiji. |
|  | Contact mode | Initial contact was made with every school in Fiji (n=912), via email, inviting their parents and children to participate. Additionally, a targeted in-person recruitment effort was completed by the research team, with face-to-face visits to 61 schools to discuss the survey with the school principal, asking them to distribute and encourage participation from their parents and students. |
|  | Advertising the survey | Survey links and QR codes were distributed to schools via email for caregivers to access the online survey. Videos advertising the survey were created to promote the study on school social media accounts, and were available in iTaukei/Fijian, Fijian-Hindi and English. |
| Survey administration | Web/E-mail | Web-based survey with automatic response capture using REDCap. |
|  | Context | Survey distributed through schools and educational networks in Fiji. |
|  | Mandatory/voluntary | Participation was voluntary. |
|  | Incentives | Principals were offered a school-level report summarising the results, while caregivers could enter a draw to win a $100 voucher by completing both caregiver and child components. |
|  | Time/Date | Survey data were collected between July 2023 and April 2024. |
|  | Randomisation of items or questionnaires | Questions were ordered by demographics, knowledge of recommendations with randomised response options, and then questions relating to attitudes (importance of an outcome, for example health, and its perceived relationship to meeting recommendations). Children were asked questions in the same order, excluding demographics. All attitude questions can be found in Table 1, 2 and 3. |
|  | Adaptive questioning | Adaptive questioning, skip logic and calculation fields reduced survey length. |
|  | Number of Items | The number of items was 80 for caregivers, 41 for children aged 5-8 years, and 65 for children aged 9-17 years. |
|  | Number of screens (pages) | The survey spanned 13, five, and eight pages respectively for caregivers, children aged 5-8, and children aged 9-17. |
|  | Completeness check | All fields were mandatory, prompting participants to complete missed responses before proceeding. |
|  | Review step | The respondents were not able to go back, review and change their answers and were not displayed a summary of their responses. |
| Response rates | Unique site visitor | Not collected. |
|  | View rate (Ratio of unique survey visitors/unique site visitors) | Not collected. |
|  | Participation rate (Ratio of unique visitors who agreed to participate/unique first survey page visitors) | Unique site visitors, survey view rates and participation rates were not collected. |
|  | Completion rate (Ratio of users who finished the survey/users who agreed to participate) | To report the survey response rate, the completion rate (the ratio of users who finished the survey to the users who agreed to participate) was captured. The survey completion rate was 42% (699 caregiver-child dyads completed out of 1663 who consented). |
| Preventing multiple entries from the same individual | Cookies used | Not used. |
|  | IP check | Not used. |
|  | Log file analysis | Not used. |
|  | Registration | Not used. |
| Analysis | Handling of incomplete questionnaires | Surveys were retained in the dataset regardless of their submission time. No statistical corrections for missing data were conducted. Cautious exclusions were implemented for perceived contamination risks or improbable response patterns. |
|  | Questionnaires submitted with an atypical timestamp | Surveys were retained in the dataset regardless of their submission time. |
|  | Statistical correction | No statistical corrections for missing data were conducted, however duplicate entries from the same individual were identified by matching contact details and manually merged, prioritising the most complete version based on the earlier commencement date. This merging only occurred if the caregiver had reached the section viewing recommendation definitions, to avoid potential contamination or carry-over effects on subsequent knowledge and physical activity/screen time duration items. |

This checklist has been modified from Eysenbach G. Improving the quality of Web surveys: the Checklist for Reporting Results of Internet E-Surveys (CHERRIES). J Med Internet Res. 2004 Sep 29;6(3):e34 [erratum in J Med Internet Res. 2012; 14(1): e8.]. Article available at [https://www.jmir.org/2004/3/e34](https://www.jmir.org/2004/3/e34/)/; erratum available <https://www.jmir.org/2012/1/e8/>. Copyright ©Gunther Eysenbach. Originally published in the [Journal of Medical Internet](http://www.jmir.org) Research, 29.9.2004 and 04.01.2012.

This is an open-access article distributed under the terms of the Creative Commons Attribution License (<https://creativecommons.org/licenses/by/2.0/>), which permits unrestricted use, distribution, and reproduction in any medium, provided the original work, first published in the Journal of Medical Internet Research, is properly cited.

Table A2. Methodology Details

| Physical Activity:  “On an average weekday/weekend day (over a 24-hour period), how much time does your child participate in sport, physical activity and active play?” |
| --- |
| Sedentary Recreational Screen Time: “On an average weekday/weekend day (24-hour period), how much time does your child spend using any electronic screen device such as a smartphone, tablet, video game, or watch television or movies, videos on the internet while they were sitting or lying down?” |
| Videos Advertising Survey:  English: https://www.youtube.com/watch?v=_MG1qTCeF6w  iTaukei/Fijian: https://www.youtube.com/watch?v=FcDwK5Q9EBM  Fijian-Hindi: https://www.youtube.com/watch?v=_3w4kpKKQIE |
| Videos Explaining screen time and physical activity guidelines: MVPA Explainer: https://youtu.be/jekl8MXWp1k  screen time Explainer: https://youtu.be/VjQxSGK3fo0 |

Supplementary File B:

Table B1. Caregivers’ motivation to comply with certain people

| Caregivers Motivation to Comply with | Total | Do no care  n (%) | Neither  n (%) | Care  n (%) | Not applicable n (%) |
| --- | --- | --- | --- | --- | --- |
| Partner | 958 | 27 (3) | 47 (5) | 816 (85) | 68 (7) |
| Family | 957 | 52 (5) | 56 (63) | 828 (87) | 21 (2) |
| Friends | 957 | 221 (23) | 155 (16) | 529 (55) | 52 (6) |
| Religious Leaders | 956 | 117 (12) | 135 (14) | 668 (70) | 36 (4) |
| People on Social Media | 956 | 533 (56) | 146 (15) | 216 (23) | 61 (6) |

Table B2. Caregivers’ motivation to comply by language group (iTaukei/Fijian, Fijian Hindi, English)

|  | Population | Total | Do not care  n (%) | Neither  n (%) | Care  n (%) | Not applicable  n (%) |
| --- | --- | --- | --- | --- | --- | --- |
| Person | iTaukei / Fijian | 396 | 11 (3) | 24 (6) | 338 (85) | 23 (6) |
| Partner | Fijian Hindi | 345 | 8 (2) | 12 (3) | 301 (87) | 24 (7) |
|  | English | 210 | 7 (3) | 11 (5) | 172 (82) | 20 (10) |
|  | iTaukei / Fijian | 396 | 14 (4) | 28 (7) | 348 (88) | 6 (2) |
| Family | Fijian Hindi | 344 | 16 (5) | 10 (3) | 311 (90) | 7 (2) |
|  | English | 210 | 21 (10) | 17 (8) | 164 (78) | 8 (4) |
|  | iTaukei / Fijian | 396 | 73 (18) | 64 (16) | 241 (61) | 18 (5) |
| Friends | Fijian Hindi | 344 | 88 (26) | 53 (15) | 181 (53) | 22 (6) |
|  | English | 210 | 58 (28) | 36 (17) | 104 (50) | 13 (6) |
|  | iTaukei / Fijian | 396 | 25 (6) | 53 (13) | 308 (77) | 13 (2) |
| Religious Leaders | Fijian Hindi | 343 | 52 (15) | 44 (13) | 235 (69) | 12 (3) |
|  | English | 210 | 38 (18) | 36 (17) | 125 (60) | 11 (5) |
|  | iTaukei / Fijian | 396 | 198 (50) | 71 (18) | 102 (26) | 25 (6) |
| People on Social Media | Fijian Hindi | 343 | 211 (62) | 39 (11) | 74 (22) | 19 (6) |
|  | English | 210 | 120 (57) | 33 (16) | 40 (19) | 17 (8) |

Table B3. Caregivers’ motivation to comply by region (urban, rural, remote & very remote)

| Person | Population | Total | Do not care  n (%) | Neither  n (%) | Care  n (%) | Not applicable  n (%) |
| --- | --- | --- | --- | --- | --- | --- |
| Partner | Urban | 641 | 19 (3) | 30 (5) | 542 (85) | 50 (8) |
|  | Rural | 144 | 6 (4) | 8 (6) | 119 (83) | 11 (8) |
|  | Remote & Very Remote | 170 | 2 (1) | 9 (5) | 152 (89) | 7 (4) |
| Family | Urban | 640 | 38 (6) | 39 (6) | 550 (86) | 13 (2) |
|  | Rural | 144 | 9 (6) | 9 (6) | 122 (85) | 4 (3) |
|  | Remote & Very Remote | 170 | 5 (3) | 8 (5) | 153 (90) | 4 (2) |
| Friends | Urban | 640 | 148 (23) | 112 (18) | 344 (54) | 36 (6) |
|  | Rural | 144 | 38 (26) | 22 (15) | 78 (54) | 6 (4) |
|  | Remote & Very Remote | 170 | 34 (20) | 21 (12) | 106 (62) | 9 (5) |
| Religious Leaders | Urban | 639 | 88 (14) | 101 (16) | 426 (67) | 24 (4) |
|  | Rural | 144 | 14 (10) | 15 (10) | 110 (76) | 5 (3) |
|  | Remote & Very Remote | 170 | 15 (9) | 19 (11) | 129 (76) | 7 (4) |
| People on Social Media | Urban | 639 | 366 (57) | 104 (16) | 127 (20) | 42 (7) |
|  | Rural | 144 | 77 (53) | 16 (11) | 42 (29) | 9 (6) |
|  | Remote & Very Remote | 170 | 88 (52) | 26 (15) | 46 (27) | 10 (6) |

### Table B4. Caregivers trust in institutions/groups to provide health information by Language Group (iTaukei/Fijian, Fijian Hindi, English)

|  | Population | Total | Disagree  n (%) | Neither agree nor disagree  n (%) | Agree  n (%) |
| --- | --- | --- | --- | --- | --- |
| Ministry of Health |  |  |  |  |  |
|  | iTaukei / Fijian | 406 | 14 (3) | 31 (8) | 361 (89) |
|  | Fijian Hindi | 357 | 10 (3) | 15 (4) | 332 (93) |
|  | English | 217 | 10 (5) | 24 (11) | 183 (84) |
| Ministry of Education |  |  |  |  |  |
|  | iTaukei / Fijian | 406 | 7 (2) | 31 (8) | 368 (91) |
|  | Fijian Hindi | 357 | 7 (2) | 19 (5) | 331 (93) |
|  | English | 214 | 11 (5) | 17 (8) | 186 (87) |
| The Ministry of Youth & Sports |  |  |  |  |  |
|  | iTaukei / Fijian | 404 | 16 (4) | 54 (13) | 334 (83) |
|  | Fijian Hindi | 357 | 13 (4) | 41 (11) | 303 (85) |
|  | English | 216 | 10 (5) | 31 (14) | 175 (81) |
| Teachers and Schools |  |  |  |  |  |
|  | iTaukei / Fijian | 406 | 6 (1) | 33 (8) | 367 (90) |
|  | Fijian Hindi | 357 | 9 (3) | 15 (4) | 333 (93) |
|  | English | 217 | 5 (2) | 24 (11) | 188 (87) |
| Physical activity and sports programs |  |  |  |  |  |
|  | iTaukei / Fijian | 406 | 2 (0) | 35 (9) | 369 (93) |
|  | Fijian Hindi | 357 | 6 (2) | 20 (6) | 331 (93) |
|  | English | 216 | 10 (5) | 18 (8) | 188 (87) |
| Community Leaders |  |  |  |  |  |
|  | iTaukei / Fijian | 405 | 36 (9) | 92 (23) | 277 (68) |
|  | Fijian Hindi | 357 | 50 (14) | 82 (23) | 225 (63) |
|  | English | 216 | 30 (14) | 48 (22) | 138 (64) |
| Religious institutions |  |  |  |  |  |
|  | iTaukei / Fijian | 405 | 25 (6) | 68 (17) | 312 (77) |
|  | Fijian Hindi | 357 | 39 (11) | 81 (23) | 237 (66) |
|  | English | 216 | 29 (13) | 45 (21) | 142 (66) |
| Social Media (eg. Facebook & Instagram) |  |  |  |  |  |
|  | iTaukei / Fijian | 406 | 103 (25) | 121 (30) | 182 (45) |
|  | Fijian Hindi | 357 | 73 (20) | 98 (27) | 186 (52) |
|  | English | 215 | 55 (26) | 52 (24) | 108 (50) |

### Table B5: Caregivers trust in institutions/groups to provide health information by Region (Urban, Rural, Remote & Very Remote)

|  | Population | Total | Disagree  n (%) | Neither agree nor disagree  n (%) | Agree  n (%) |
| --- | --- | --- | --- | --- | --- |
| Ministry of Health |  |  |  |  |  |
|  | Urban | 664 | 29 (4) | 49 (7) | 586 (88) |
|  | Rural | 149 | 2 (1) | 10 (7) | 137 (92) |
|  | Remote & Very Remote | 171 | 3 (2) | 12 (7) | 156 (91) |
| Ministry of Education |  |  |  |  |  |
|  | Urban | 664 | 18 (3) | 48 (7) | 598 (90) |
|  | Rural | 149 | 2 (1) | 8 (5) | 139 (93) |
|  | Remote & Very Remote | 171 | 5 (3) | 11 (6) | 155 (91) |
| The Ministry of Youth & Sports |  |  |  |  |  |
|  | Urban | 662 | 9 (4) | 89 (13) | 544 (82) |
|  | Rural | 148 | 6 (4) | 16 (11) | 126 (85) |
|  | Remote & Very Remote | 171 | 4 (2) | 23 (13) | 144 (84) |
| Teachers and Schools |  |  |  |  |  |
|  | Urban | 664 | 14 (2) | 52 (8) | 598 (90) |
|  | Rural | 149 | 3 (2) | 7 (5) | 139 (93) |
|  | Remote & Very Remote | 171 | 3 (2) | 14 (8) | 154 (90) |
| Physical activity and sports programs |  |  |  |  |  |
|  | Urban | 663 | 16 (2) | 51 (8) | 596 (90) |
|  | Rural | 149 | 1 (1) | 9 (6) | 139 (93) |
|  | Remote & Very Remote | 171 | 1 (1) | 13 (8) | 157 (92) |
| Community Leaders |  |  |  |  |  |
|  | Urban | 662 | 80 (12) | 148 (22) | 434 (66) |
|  | Rural | 149 | 19 (3) | 38 (5) | 92 (62) |
|  | Remote & Very Remote | 171 | 17 (10) | 40 (23) | 114 (67) |
| Religious institutions |  |  |  |  |  |
|  | Urban | 662 | 70 (11) | 137 (21) | 455 (69) |
|  | Rural | 149 | 12 (8) | 27 (18) | 110 (74) |
|  | Remote & Very Remote | 171 | 11 (6) | 33 (19) | 127 (74) |
| Social Media (eg. Facebook & Instagram) |  |  |  |  |  |
|  | Urban | 662 | 152 (23) | 182 (27) | 328 (50) |
|  | Rural | 149 | 36 (24) | 46 (31) | 67 (45) |
|  | Remote & Very Remote | 151 | 43 (28) | 48 (32) | 60 (40) |

Table B6. Children aged 5-8 years’ motivation to comply with certain people

| Person | Total | No, do not care  n (%) | Yes, I do care  n (%) |
| --- | --- | --- | --- |
| Family | 207 | 7 (3) | 200 (96) |
| Friends | 206 | 49 (24) | 157 (76) |
| Religious Leaders | 206 | 45 (22) | 161 (78) |
| Sports Champions | 206 | 49 (24) | 157 (76) |
| Teachers | 206 | 3 (2) | 203 (98) |

Table B7. Children 5-8 years motivation to comply by language group (iTaukei/Fijian, Fijian Hindi, English)

| Person | Population | Total | No, do not care  n (%) | Yes, I do care  n (%) |
| --- | --- | --- | --- | --- |
| Family | iTaukei / Fijian | 80 | 2 (3) | 78 998) |
|  | Fijian Hindi | 82 | 3 (4) | 79 (96) |
|  | English | 43 | 2 (5) | 41 (95) |
| Friends | iTaukei / Fijian | 60 | 17 (28) | 43 (72) |
|  | Fijian Hindi | 61 | 19 (31) | 42 (69) |
|  | English | 43 | 13 (30) | 30 (70) |
| Religion | iTaukei / Fijian | 80 | 9 (11) | 71 (89) |
|  | Fijian Hindi | 81 | 23 (28) | 30 (70) |
|  | English | 43 | 13 (30) | 30 (70) |
| Sport | iTaukei / Fijian | 80 | 13 (16) | 67 (84) |
|  | Fijian Hindi | 81 | 26 (32) | 55 (68) |
|  | English | 43 | 10 (23) | 33 (77) |
| Teachers | iTaukei / Fijian | 80 | 1 (1) | 79 (99) |
|  | Fijian Hindi | 81 | 1 (1) | 80 (99) |
|  | English | 43 | 1 (2) | 42 (98) |

Table B8. Children 5-8 years motivation to comply by region (urban, rural, remote & very remote)

| Person | Population | Total | No, do not care  n (%) | Yes, I do care  n (%) |
| --- | --- | --- | --- | --- |
| Family | Urban | 134 | 5 (4) | 129 (96) |
|  | Rural | 26 | 2 (8) | 24 (92) |
|  | Remote & Very Remote | 45 | 0 (0) | 45 (100) |
| Friends | Urban | 133 | 36 (27) | 97 (73) |
|  | Rural | 26 | 7 (27) | 19 (73) |
|  | Remote & Very Remote | 45 | 6 (13) | 39 (87) |
| Religion | Urban | 133 | 35 (26) | 98 (74) |
|  | Rural | 26 | 4 (15) | 22 (85) |
|  | Remote & Very Remote | 45 | 6 (13) | 39 (87) |
| Sport | Urban | 133 | 38 (29) | 95 (71) |
|  | Rural | 26 | 4 (15) | 22 (85) |
|  | Remote & Very Remote | 45 | 7 (16) | 38 (84) |
| Teachers | Urban | 133 | 3 (2) | 130 (98) |
|  | Rural | 26 | 0 (0) | 26 (100) |
|  | Remote & Very Remote | 45 | 0 (0) | 45 (100) |

Table B9. Children aged 9-17 years’ motivation to comply with certain people

| Person | Total | No, do not care  n (%) | Yes, I do care  n (%) |
| --- | --- | --- | --- |
| Family | 523 | 20 (4) | 503 (96) |
| Friends | 523 | 152 (29) | 371 (71) |
| Religious Leaders | 522 | 90 (17) | 432 (83) |
| Sports Champions | 522 | 126 (24) | 396 (76) |
| Teachers | 522 | 12 (2) | 510 (98) |
| People on Social Media | 522 | 345 (66) | 177 (34) |

Table B10. Children 9-17 years motivation to comply by language group (iTaukei/Fijian, Fijian Hindi, English)

| Person | Population | Total | No, do not care  n (%) | Yes, I do care  n (%) |
| --- | --- | --- | --- | --- |
| Family | iTaukei / Fijian | 227 | 5 (2) | 222 (98) |
|  | Fijian Hindi | 183 | 8 (4) | 175 (96) |
|  | English | 109 | 7 (6) | 102 (94) |
| Friends | iTaukei / Fijian | 227 | 67 (30) | 160 (70) |
|  | Fijian Hindi | 183 | 49 (27) | 134 (73) |
|  | English | 109 | 35 (32) | 74 (68) |
| Religion | iTaukei / Fijian | 226 | 25 (11) | 201 (89) |
|  | Fijian Hindi | 183 | 38 (21) | 145 (79) |
|  | English | 109 | 27 (25) | 82 (75) |
| Sport | iTaukei / Fijian | 226 | 50 (22) | 176 (78) |
|  | Fijian Hindi | 183 | 42 (23) | 141 (77) |
|  | English | 109 | 33 (30) | 76 (70) |
| Teachers | iTaukei / Fijian | 226 | 1 (0) | 225 (100) |
|  | Fijian Hindi | 183 | 5 (3) | 178 (97) |
|  | English | 109 | 5 (5) | 104 (95) |
| People on Social | iTaukei / Fijian | 226 | 144 (64) | 82 (36) |
|  | Fijian Hindi | 183 | 119 (65) | 64 (35) |
|  | English | 109 | 79 (72) | 30 (28) |

Table B11. Children 9-17 years motivation to comply by region (urban, rural, remote & very remote)

| Person | Population | Total | No, do not care  n (%) | Yes, I do care  n (%) |
| --- | --- | --- | --- | --- |
| Family | Urban | 343 | 13 (4) | 330 (96) |
|  | Rural | 78 | 4 (5) | 74 (95) |
|  | Remote & Very Remote | 101 | 3 (3) | 98 (97) |
| Friends | Urban | 343 | 95 (28) | 248 (72) |
|  | Rural | 78 | 29 (37) | 49 (63) |
|  | Remote & Very Remote | 101 | 28 (28) | 73 (72) |
| Religion | Urban | 342 | 65 (19) | 277 (81) |
|  | Rural | 78 | 13 (17) | 65 (83) |
|  | Remote & Very Remote | 101 | 12 (12) | 89 (88) |
| Sport | Urban | 339 | 82 (24) | 257 (76) |
|  | Rural | 78 | 14 (18) | 64 (82) |
|  | Remote & Very Remote | 101 | 27 (27) | 74 (73) |
| Teachers | Urban | 342 | 8 (2) | 334 (98) |
|  | Rural | 78 | 2 (3) | 76 (97) |
|  | Remote & Very Remote | 101 | 2 (2) | 99 (98) |
| People on Social | Urban | 342 | 235 (69) | 107 (31) |
|  | Rural | 78 | 44 (56) | 34 (44) |
|  | Remote & Very Remote | 101 | 65 (64) | 36 (36) |
